# Biopsychosocial Aspects of Atypical Odontalgia

**DOI:** 10.1155/2013/413515

**Published:** 2013-03-05

**Authors:** A. Ciaramella, M. Paroli, L. Lonia, M. Bosco, P. Poli

**Affiliations:** ^1^Pain Therapy Unit, Department of Oncology, Azienda Ospedaliero-Universitaria Pisana, Via Roma 67, 56127 Pisa, Italy; ^2^Department of Head and Neck, Azienda Ospedaliero-Universitaria Pisana, Via Roma 67, 56127 Pisa, Italy; ^3^Department of Dentistry, University of Pavia, Via Ferrara 1, 27100 Pavia, Italy

## Abstract

*Background*. A few studies have found somatosensory abnormalities in atypical odontalgia (AO) patients. The aim of the study is to explore the presence of specific abnormalities in facial pain patients that can be considered as psychophysical factors predisposing to AO. *Materials and Methods*. The AO subjects (*n* = 18) have been compared to pain-free (*n* = 14), trigeminal neuralgia (*n* = 16), migraine (*n* = 17), and temporomandibular disorder (*n* = 14). The neurometer current perception threshold (CPT) was used to investigate somatosensory perception. Structured clinical interviews based on the DSM-IV axis I and DSM III-R axis II criteria for psychiatric disorders and self-assessment questionnaires were used to evaluate psychopathology and aggressive behavior among subjects. *Results*. Subjects with AO showed a lower A**β**, A**δ**, and C trigeminal fiber pain perception threshold when compared to a pain-free control group. Resentment was determined to be inversely related to A**β** (rho: 0.62, *P* < 0.05), A**δ** (rho: 0.53, *P* < 0.05) and C fibers (rho: 0.54, *P* < 0.05), and depression was inversely related with C fiber (rho: 0.52, *P* < 0.05) perception threshold only in AO subjects. *Conclusion*. High levels of depression and resentment can be considered predictive psychophysical factors for the development of AO after dental extraction.

## 1. Introduction

Atypical Odontalgia (AO) is a persistent pain condition located in the teeth and jaws. It has been described as a persistent neuropathic pain that may be initiated after the deafferentiation of trigeminal nerve fibers following a root canal treatment, an apicoectomy, or a tooth extraction, or it may be of idiopathic origin [1]. The terminology and specific criteria for its classification remain a matter of discussion [2]. The International Headache Society [3] considers AO to be a type of persistent, idiopathic, orofacial pain that is often difficult to diagnose because it is associated with a lack of clinical and radiographic abnormalities. Laboratory investigations, including X-rays of the face, jaws and teeth, do not indicate any relevant abnormalities. In the case of a tooth extraction, the pain is found in the edentate area and usually extends to the other adjacent facial structures. Several criteria for the diagnosis of AO have been suggested [4, 5]. 

A few studies have found somatosensory abnormalities in AO patients [6–8]. These sensory modifications were located intraoral on the site of the treated tooth, suggesting a disturbance of the central processing or craniofacial information carried by the trigeminal nerve [9].

However, a lack of apparent physical causes has led some researchers to associate AO with abnormal psychological states. A depressed mood and somatization are often related to the experience of chronic pain, but no AO-pain-prone personality type has been identified [10–13]. Consequently, a “yellow flags” chronic orofacial pain screening for psychosocial risk factors has been proposed [14], and stress has been identified as a possible pathophysiological contributor that underlines depression and facial pain [15]. 

The purpose of this study is to explore the presence of specific abnormalities in facial pain patients that can be considered as psychophysical factors predisposing to AO. 

## 2. Methods

### 2.1. Design

**Figure 1:**
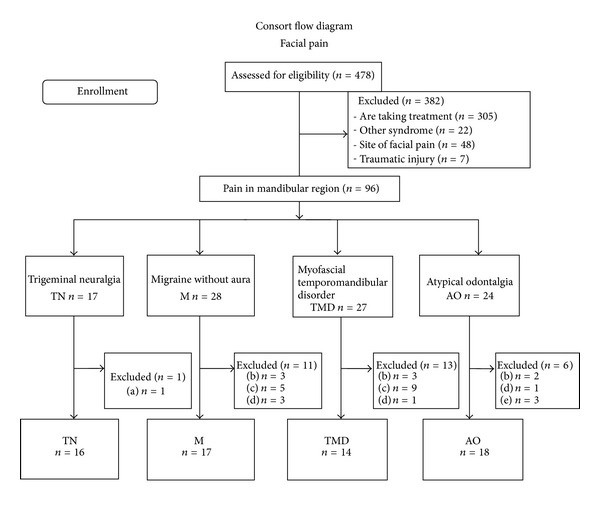
(a) Neurovascular conflict, (b) facial pain comorbidity, (c) musculoskeletal pain comorbidity, (d) declined to participate, and (e) not according with criteria.

A case-control study was used to compare AO patients with control groups that presented with other forms of facial pain and with a pain-free control group (PF). The noncase chronic facial pain groups were (1) trigeminal neuralgia (TN), (2) migraine without aura (M), and (3) myofascial temporomandibular disorder (TMD) (Figure 1). In the TMD group, the concerns focused on the clinical disturbances affecting the masticator muscles. The local ethics committee approved all procedures, and written informed consent was obtained from each subject prior to inclusion in the study. 

The assessment was performed in two sessions with a mean of 1 hour between sessions:

- neurometric test (current perception threshold, CPT),
- psychosocial interview.

### 2.2. Sample

Consecutive subjects with facial pain were recruited from the Pain Therapy Unit of Santa Chiara Hospital in Pisa and the Dentistry Clinic at the University of Pisa. All facial pain subjects were screened before to be sent to the psychophysics pain laboratory located in the Pain Therapy Unit. Subjects were examined using a clinical tool based on the ICDH II criteria of the International Headache Society [3] and were screened for AO, TN, M, and TMD. Subjects were included in the study if they reported pain in the mandibular region. The following criteria were used to exclude subjects: age under 18 years; history of neuromuscular or skeletal disease; history of other TMD or stomatognathic diseases; nonsteroidal anti-inflammatory drugs, corticosteroids, muscle relaxants, benzodiazepine, or tricyclic antidepressants continuous treatments; acute and/or chronic traumatic injury; metabolic disease; drug abuse; dental or TMD treatment in the previous 6 months; the presence of more than one facial pain. Patients were also excluded if an MRI showed a neurovascular conflict related to pain. Subjects with a history of migraine headaches were selected only if they did not experience auras and did not have comorbid head and facial pain. Clinical facial pain patients were diagnosed with criteria from the International Headache Society [3] (Tables 1 and 2). Patients with temporomandibular disorder (TMD) were identified using Dworkin and LeResche's criteria for temporomandibular disorders (axis I) and muscle disorders (group I) (Table 3) [16]. AO patients were identified using the Marbach criteria (Table 4) [17].

All clinical examinations were completed by the same trained operator and performed according to the RDC/TMD axis I criteria. The restrictive exclusion criteria and necessary presence of a unique form of facial pain resulted in a small number of selected subjects relative to the total sample. The high number of pain comorbid syndrome and the narrowness of the inclusion criteria allowed us to include only 65 of the 478 subjects with facial pain initially selected. Three hundred eighty-two facial pain patients in database of electronic medical record were excluded mainly because of being under drug treatment. Only 96 were approached personally, and, using the previous criteria, just a total of 65 subjects were selected (check the flow chart). The pain-free control group (PF) consisted of volunteers from the medical and nursing staff of the pain therapy and dentistry clinic. All volunteers had a history of surgical procedure or extraction of at least one tooth without consequential persistent pain. 

The subjects selected for the study were taking anti-inflammatory medication as needed, and the last administration was more than 6 hours before assessment with the current perception threshold (CPT) test. 

A structured interview was conducted. The interview included demographic, family, and social data as well as any distressing events that the individuals had experienced in the last 6 months. Information regarding the patient's lifetime medical conditions was also recorded. 

### 2.3. Psychophysical Sensory Evaluation

The neurometer CPT is a transcutaneous electrical stimulator that delivers sinusoidal electrical stimuli via surface electrodes at frequencies of 5 Hz, 250 Hz, and 2000 Hz at a current intensity range of .01 to 9.99 mA [18]. This technique is a semiquantitative method used to quantify sensory nerve dysfunctions in patients with neuropathic pain [19–21].

Several studies have demonstrated the selective fibers excitation of CPT [22, 23]. These studies reported that 5 Hz CPT measures correlated with small-fibers C, 250-Hz CPT measures correlated with A*δ* fibers, and 2000 Hz CPT measures correlated with large diameter fibers A*β* [24].

The transmitting electrodes were placed on the anterior region of the tragus bilaterally, and the electrical stimuli (registration) were started in the unaffected (pain-free) side. The tragus was selected as the landmark of the mandibular branch of the trigeminal nerve. We employed a total scoring derived from the mean sum of the CPT threshold on each tragus side of 2000, 250, and 5 Hz using the formula L + R/2 (left + right/2).

### 2.4. Psychopathology Assessment

The Italian adaptation of the Irritability Depression Anxiety Scale (IDAS) [25, 26] is a 14-item self-administered assessment that includes 4 items to assess irritability, 5 items to measure anxiety, and 5 items to evaluate depression. The IDAS is a validated instrument capable of distinguishing between depressive and anxiety disorders [27]. It has been used to screen for depression in patients with oral dysesthesia [28] and to measure outcomes during rehabilitation after a stroke [29]. The investigation of psychopathology on this scale is different from other psychopathological scales used in this study. IDAS scale investigates depression not only with symptoms of negative mood but also using the absence of positive mood. The irritability according to Snaith et al. [25] is different by aggression, violent outbursts, hostility, bad temper, anger, intolerance, and so on. We also investigated the hostility using SCL-90-R and all profile of aggressive behavior using another appropriate questionnaire. The Symptom Checklist-90-R (SCL-90-R) contains 90 items that measure 9 primary symptom dimensions: somatization (SOM), obsessive-compulsive (OC), interpersonal sensitivity (IS), depression (DEP), anxiety (ANX), hostility (HOS), phobic anxiety (PHOB), paranoid ideation (PAR), and psychoticism (PSY). The SCL-90-R is an important and valid instrument used to assess TMD axis II disorders according to the RDC/TMD [30, 31]. 

The Mini International Neuropsychiatric Interview (MINI) is a structured diagnostic interview for lifetime DSM-IV axis I disorders. It relies on ICD-10 criteria [32]. In addition to being easy to use and brief, it is a valid and reliable tool for the exploration of psychiatric disorders among subjects with pain [33].

The SCID-II Personality Questionnaire is a screening tool developed for the assessment of personality disorders. Several studies have reported that the SCID for the DSM-III-R and DSM-IV is valid and reliable. This study used the DSM-III-R version [34–36].

### 2.5. Behavioral Assessment

The BDHI (Buss-Durkee Hostility Inventory) [37, 38] is a 75-item self-assessment questionnaire that investigates aggressive behavior. Patients respond to each BDHI item using a true or false format. The following 8 aggressive behavior dimensions were investigated: assault, indirect hostility, irritability, negativism, resentment, suspicion, verbal hostility, and guilt.

### 2.6. Pain Assessment

Pain assessment was conducted using the Italian Pain Questionnaire (IPQ) [39]. The IPQ is derived from the McGill Pain Questionnaire (MPQ) and uses the factorial structure proposed by Melzack and Torgerson [40]. The IPQ was built *ex novo* using dimensions and structure of the MPQ; it was validated by Italian population [39]. The structure is made up of three factors or classes (Sensorial, Affective, and Evaluative). Pain intensity is assessed by a 0–10 Visual Analogue Scale (VAS) [41].

The Multidimensional Pain Inventory (MPI) [42] is a comprehensive instrument used to assess a number of dimensions of the chronic pain experience, including pain intensity, emotional distress, cognitive and functional adaptation, and social support. It is one of the best instruments available to assess the overall adjustment of chronic pain patients and the outcomes of treatment interventions. The utility of the MPI has been demonstrated in samples of patients with various chronic pain syndromes. The MPI is a validated instrument used to assess RDC/TMD axis II disorders [43–45].

## 3. Statistical Analysis

The small size of facial pain groups required the use of nonparametric analysis in the comparison of all investigated dimensions among groups. Non-parametric analyses were performed to distinguish differences in behavior, psychopathology, and current thresholds by applying the Kruskal-Wallis and Mann-Whitney tests. Spearman rank correlation analysis was used to investigate a possible relationship among the dimensions of each psychopathological test and the CPT thresholds in the total sample, in painful conditions with the exception of the AO individuals and the AO group alone. Logistic regression (stepwise) was performed to investigate which dimensions of BDHI, SCL 90-R, and IDAS were associated to AO group compared to other pain syndromes and pain-free group. The dimensions obtained from that template were the independent variables in the linear regression; threshold of each fiber of CPT was the dependent variable, considering independent variables as potential predictors for AO individuals or total pain sample (without pain free). Differences in the frequency of psychiatric disorders among various diagnostic pain groups were assessed with *χ* ^2^ analysis using Fisher's exact test for a small sample. Data are presented as the mean ± SD with a level of significance at *P* < 0.05.

## 4. Results

### 4.1. Clinical Variables and Current Perception Threshold (CPT)

The 5 groups were made up of 18 individuals with AO, 16 individuals with TN, 17 individuals with M, 14 individuals with TMD, and 14 PF individuals. No relationship was found between the site of pain and the CPT of specific nerve fibers (Table 5(b)). Age did not correlate with CPT (Spearman rho correlation). 

The CPT test revealed that the AO group elicited a measure of stimulus perception on both sides of the A*β* fibers at 2000 Hz. This is lower than all other groups except the TN group (Table 5(a)). The AO group also demonstrated hyperactivation of the A*δ* fiber on the right side but not on left and with a lower threshold than all other groups (Table 5(a)). The CPT of unmyelinated C fibers was the same in the AO and TN groups, but not in the other pain groups (Tables 5(a) and 5(b)). If we consider the mean of the sum of scoring of the bilateral CPT fibers threshold (L + R/2), we found that the AO group displayed a lower threshold of A*β*, A*δ*, and C fibers than the PF groups did (Table 6).

### 4.2. Differences among Groups in Psychological Dimensions and Psychiatric Disorders

*Behavior.* In terms of aggressive behavior (BDHI), the AO patient group demonstrated higher levels of resentment than other groups did (Table 7). A logistic regression analysis with 95% confidence interval (CI) was performed to investigate the association between the aggressive behavioral dimensions and AO. Resentment was found to be associated with AO (*P* = 0.001 with expectation degree of 1.10). 

*Psychopathology.* The AO group demonstrated higher levels of depression on the IDAS than either the PF or TMD groups (Table 7). Again, a logistic regression analysis with 95% CI indicated that depression is more strongly associated with AO than it is with any of the other groups (*P* = 0.02; expectation degree of 1.30).

No differences were found in most of the psychopathology measures investigated with the SCL 90-R. The only exception was somatization, which was higher in the TMD group (*χ* ^2^ = 7.49; *P* < 0.05). According to the SCL-90-R, somatization was higher in all pain groups, except the AO group, than in pain-free subjects (Table 7). The AO group scored higher for psychoticism on the SCL-90-R than the pain-free subjects (Table 7). The depression dimension of the SCL-90-R was strongly correlated with the identification of depression on the IDAS (Spearman Rank *z* value = 3.53; *P* = 0.0004). A significant correlation was found between resentment on the BHDI and the hostility dimension of the SCL-90-R (Spearman rank *z* value = 2.41; *P* = 0.015). 

Because of the small number of subjects in each group, Fisher's exact test cannot be used for the analysis of contingency or to identify differences in the frequency of psychiatric disorders between groups (based on the MINI interview). Statistical analysis was applied only to current depressive episode data and found that a current major depressive episode (CMDE) was more frequent in the AO group than in other groups (*χ* ^2^ = 11.12; *P* < 0.05).

The identification of personality disorders using SCID II according to DSM III-R criteria showed a difference between groups for some disorders. Avoidant disorder was more frequent in the AO group than in other groups (*χ* ^2^ = 9.72; *P* < 0.05); obsessive-compulsive personality disorder was more frequent in the M group (*χ* ^2^ = 12.83; *P* < 0.01) than in other groups; paranoid disorder was more frequent in the TN group (*χ* ^2^ = 10.32; *P* < 0.05) than in other groups.

### 4.3. Differences among Groups in Pain Experience

The TN group experienced more intense pain (VAS measure) than other groups (Kruskal-Wallis analysis *χ* ^2^ = 24.27; *P* < 0.001). According to the MPI, the AO group reported fewer solicitous and distracting responses from family members than other groups (*χ* ^2^ = 20.81; *P* < 0.001 and *χ* ^2^ = 11.93; *P* < 0.01). The TMD group received less support from family than the other groups (*χ* ^2^ = 16.33; *P* < 0.01). The AO group scored lowest on the “activities away from home” section of the MPI (*χ* ^2^ = 10.51; *P* < 0.05).

### 4.4. Correlation between Aggressive Behavior, Pain, Psychopathological Dimensions, and CPT

Depression and resentment were positively correlated in all pain subjects (Spearman rho coefficient 0.33; *P* < 0.05) and were even more strongly correlated in AO subjects (Spearman rho coefficient 0.62; *P* < 0.01). 

In a linear regression model analysis, depression, as measured on the IDAS, was also determined to be a predictor of low threshold C fibers in all pain subjects (*F* = 5.38; *P* = 0.024) and in the AO group (*F* = 9.10; *P* = 0.009). Resentment, as measured on the BDHI, was found to be a predictor of a low perception threshold of A*β* (*F* = 5.65; *P* = 0.032) and A*δ* (*F* = 5.53; *P* = 0.034) fibers only in the AO group; this dimension of BDHI was negatively correlated with all thresholds in the AO group. These correlations have not been found in any of the other groups (Table 8). Furthermore, the AO group showed a lower threshold of C fibers correlated with depression, another correlation that has not been found in other groups (Table 8).

No relationship was found between dimensions of the SCL-90-R and CPT. We identified a statistically significant association between the presence of axis I and axis II psychiatric disorders and a modification of CPT. We also identified a strong association between the presence of CMDE and a low CPT of a*β* and C fibers in the entire sample; a similarly strong association was found between avoidant personality disorders and a decrease in CPT, but this association was only true for the a*β* fibers (Table 9). A strong association was also observed between the presence of obsessive-compulsive personality disorders and an increase in CPT of A*β* fibers (Table 9). 

Subjects with avoidant personality disorders had a higher score on the resentment and suspicion measures of the BDHI, on the depression and hostility measures of the SCL 90-R, and on the depression and anxiety measures of the IDAS (Table 9) than subjects without this personality disorder. As previously mentioned, avoidant personality disorders and CMDE were more frequent in the AO group. No relationship was found among the dimensions of pain investigated through the MPI, IPQ, and CPT.

## 5. Discussion

Our study compared AO patients with pain-free subjects and with other facial pain patients. The psychophysics methods used in this study to investigate the trigeminal fibers have been used in other clinical and laboratory studies [46–48]. The current perception threshold tests revealed that AO patients demonstrated hyperactivation of A*β*, A*δ*, and C fibers at a lower threshold than the PF subjects. We confirm preview studies [7, 8] related to a somatosensory abnormality of the face in AO patients, thus supporting the hypothesis of other investigators that the stimulus hypersensitivity of large myelinated fibers is a dramatic alteration in the sensory processing of the somatosensory system, resulting in increased excitability, decreased inhibition, and structural reorganization [49–51].

A change in the perception of pain induced by emotion has been reported in preview studies [52, 53]. Nociception was facilitated by unpleasant pictures and inhibited by pleasant pictures [54, 55]. In our study, we investigated certain dimensions of aggressive behavior and determined that there exists a relationship between the perception threshold of current stimuli and individuals with AO. The AO subjects also displayed higher levels of resentment as they had the lowest electrical stimuli threshold of A*β*, A*δ*, and C fibers. In fact, resentment was associated with AO more than any other form of facial pain. This held true for subjects without pain, as well. Thus, we could predict that greater levels of resentment could predispose an individual to abnormal somatosensory perception. This claim is based on two findings: on the one hand, we found only in the AO groups and not in the other pain syndromes (Table 8) a negative correlation between resentment and CPT threshold perception of fibers, and on the other hand, we found out the different thresholds of all fibers of sole AO group and not in other pain groups comparing pain free individuals with a history of surgical procedure or tooth extraction without consequential persistent pain (Table 6). The presence of the variation of threshold only in AO and not in other forms of chronic pain suggests that it could be more than one predisposing factor for the chronicity, resentment and could be a predisposing factor for the onset of AO. Research on the neurobiology of aggressiveness indicates that the amygdala and midbrain are involved in patterns of aggressive behavior [56], while Siegel et al. [57] found an association between affective or defensive rage (high autonomic signs), rage and electrical or chemical stimulation of the midbrain in the periaqueductal area (PAG) and the medial hypothalamus. These findings are significant because the PAG is also involved in the modulation of pain [58, 59]. The sum of the presence of resentment and depression could very well be the two independent psychological variables that predispose an individual to atypical odontalgia via an amygdala-hypothalamus-PAG-trigeminal neurophysiological dysfunction. 

The small sample size does not allow us to draw reliable conclusions about the relationship between the presence of CMDE or avoidant personality disorder and the tendency to present with AO. What can be inferred from our data is that resentment and depression are closely linked to the presence of both avoidant personality disorder and CMDE and that these conditions affect the CPT of patients with AO (Table 5). On the basis of these results, we suggest that a biopsychosocial model can be used to predict AO.

Our research supports the assertion that psychosocial distress plays an important role and contributes to the onset of widespread pain [60]. In our study, patients with AO reported a higher number of life distressing events prior to the onset of tooth pain or root canal treatment than did the other pain groups. 

## 6. Conclusions

This research indicates that certain psychological factors determine an individual's predisposition to the development of chronic pain after a tooth extraction. The group of patients with AO demonstrated higher levels of resentment and depression than those who underwent a dental extraction but did not develop chronic pain (the PF group). These psychological dimensions are associated with an alteration in the somatosensory perception of trigeminal stimulus found only in the group with AO and not in other subjects with other forms of facial pain such as TN, TMD, or M. Stressful life events also appear to be a precipitating factor in the development of chronic pain after a tooth extraction. In fact, subjects with AO reported a high number of life distressing events in the period immediately before or in coincidence with a tooth extraction.

The most significant limitation of our study is the small number of subjects. However, it is one of the first studies to compare AO subjects with those who experience other forms of facial pain or with subjects who are pain free. 

## Figures and Tables

**Table 1:** International Headache Society criteria for migraine without aurea.

Diagnostic criteria	
(A) At least five attacks fulfilling criteria (B)–(D)	
(B) Headache attacks lasting 4–72 hours* (undertreated or unsuccessfully treated)	
(C) Headache has at least two of the following characteristics:	
(i) Unilateral location	
(ii) Pulsating quality	
(iii) Moderate or severe pain intensity	
(iv) Aggravation by or causing avoidance of routine physical activity (e.g., walking or climbing stairs)	
(D) During headache at least one of the following:	
(i) Nausea	
(ii) Photophobia and phonophobia	
(E) Not attributed to another disorder	

**Table 2:** International Headache Society criteria for trigeminal Neuralgia.

Diagnostic criteria	
(A) Paroxysmal attacks of pain lasting from a fraction of a second to 2 minutes affecting one or more divisions of trigeminal nerve and fulfilling criteria (B) and (C)	
(B) Pain has at least one of the following characteristics:	
(i) Intense, sharp, superficial, or stabbing	
(ii) Precipitated from trigger areas or by trigger factors	
(C) Attacks are stereotyped in the individual patient	
(D) There is no clinically evident neurological deficit	
(E) Not attributed to another disorder	

**Table 3:** Axis I clinical TMD conditions—Group 1.

Diagnostic criteria	
(3.a) Myofascial pain	
(A) Report of pain or ache in the jaw, temples, face, periauricular area, or inside the ear at rest or during function	
(B) Pain reported by the subject in response to palpation of 3 or more of the following muscle sites (right side and left side count as separated sites for each muscle):	
Posterior temporalis	
Middle temporalis	
Anterior temporalis	
Origin of masseter	
Body of masseter	
Insertion of masseter	
Posterior mandibular region	
Submandibular region	
Lateral pterygoid area	
Tendon of temporalis	
(3.b) Myofascial pain with limited opening	
(A) Myofascial pain as defined in (3.a)	
(B) Pain-free unassisted mandibular opening of less than 40 mm	
(C) Maximum assisted opening (passive stretch) of 5 or more mm greater than pain free unassisted opening	

**Table 4:** Atypical odontalgia: revised criteria of Marbach.

Diagnostic criteria	
(A) Pain is located in the face or described as a toothache	
(B) The pain is described as a constant dull, deep ache (less than 10% of sufferers report occasional spontaneous sharp pains that overlay the ache. Sharp pain is not essential to meet criteria)	
(C) A brief (seconds to minutes) pain free period is reported upon awakening from sleep. There are no refractory periods.	
(D) Pain develops (or continues) within one month following endodontic treatment (usually in the surface of the face but ocasionally intraorally) a location with a much lowered pain threcshold (hyperalgesia), often surrounded by a larger area with less severe hyperalgesia.	
(E) Sleep is undisturbed by pain or other phantom sensations	
(F) No radiography or laboratory test suggest other sources of pain	

**Table:** (a)

		AO			TN			M			TMD			PF			df	*χ* ^2^/*z* ^†^
		*n*	xm	sd	*n*	xm	sd	*n*	xm	sd	*n*	xm	sd	*n*	xm	sd
Sex	Male	6			6			6			2			6				
	Female	12			10			11			12			8			4	3.16
Age			63.5	12.3		57.9	14.8		47.2	18.8		39	11.9		39.2	12.2		21.76***
Side of pain	Right	7			6			7			6							
	Left	9			10			8			7						3	0.33
History of pain	Months		67.25	37.55		56.16	23.17		201.53	186		110.66	98				3	16.53***
CPT trago right (mAmp)	2000 Hz		124.31	51.47		126.68	74.63		201.37	85.93		167.21	85.93		205.35	85.26	4	11.6*
	250 Hz		30.93	17.39		41.93	29.25		55.06	37.96		59.92	48.36		71.42	41.14	4	9.87*
	5 Hz		21.25	13.82		25.87	22.39		43.81	34.96		39.21	30.55		53.78	32.94	4	10.89*
CPT trago left (mAmp)	2000 Hz		138.5	47.89		172.18	86.48		212.31	62		203.23	98.65		227.64	85.26	4	10.1*
	250 Hz		39.56	21.59		55.37	48.33		54.81	30.5		108.14	107.28		73.5	43.49	4	7.11
	5 Hz		33.12	19.9		29.56	35.11		33.93	24.22		68.92	60.22		59.21	40.54	4	10.81*
Mean of total CPT (L+R/2) (mAmp)	2000 Hz		131.4	40.82		149.43	76.15		206.84	64.12		180.23	79.92		216.5	87.7	4	14.17**
	250 Hz		35.25	17.53		48.65	38.48		54.93	31.87		84.03	68.3		72.46	39.81	4	8.41
	5 Hz		27.18	13.02		27.71	28.51		38.87	27.24		54.07	39.65		56.5	33.45	4	10.7*
Distressing events		10			4			2			1						3	18.69***

*P < 0.05; **P < 0.01; ***P < 0.001; ****P < 0.0001; ^†^(*χ* ^2^ value) Kruskal Wallis test and (*z* value) Mann-Whitney *U* analyses.

AO: atypical odontalgia; TN: trigeminal neuralgia; M: migraine; TMD: temporomandibular disorder; PF: pain free.

**Table:** (b)

		Pain side	AO			*z*	*P*	TN			*z*	*P*	M			*z*	*P*	TMD			*z*	*P*
			*n*	xm	sd			*n*	xm	sd			*n*	xm	sd			*n*	xm	sd		
	R	2000 Hz	7	130.71	22.33			6	111.83	67.77			7	222.50	106.28			6	158.33	84.35		
	L	2000 Hz	9	119.33	63.31	0.37	ns	10	135.60	80.60	0.75	ns	8	194.50	67.23	0.57	ns	7	178.71	93.38	0.57	ns
CPT trago right (mAmp)	R	250 Hz	7	31.57	14.59			6	32.66	4.03			7	67.42	42.12			6	59.00	54.23		
	L	250 Hz	9	30.44	20.16	0.63	ns	10	47.50	36.41	0.27	ns	8	49.37	33.72	0.92	ns	7	60.57	51.14	0.01	ns
	R	5 Hz	7	24.71	13.08			6	22.83	4.19			7	56.00	39.01			6	39.00	35.22		
	L	5 Hz	9	18.55	14.52	1.45	ns	10	27.70	28.51	0.65	ns	8	37.37	30.91	1.15	ns	7	40.71	31.18	0.28	ns

	R	2000 Hz	7	129.85	61.02			6	191.00	36.75			7	229.14	65.02			6	203.83	109.58		
	L	2000 Hz	9	145.22	37.27	0.79	ns	10	160.90	106.67	0.65	ns	8	209.00	55.92	0.63	ns	7	206.16	106.06	0.16	ns
CPT trago left (mAmp)	R	250 Hz	7	33.71	14.20			6	42.66	14.43			7	63.71	29.87			6	113.00	120.68		
	L	250 Hz	9	44.11	25.88	0.52	ns	10	63.00	60.04	0.48	ns	8	51.25	31.16	0.92	ns	7	103.71	112.93	0.42	ns
	R	5 Hz	7	39.71	22.54			6	19.50	10.23			7	44.42	30.78			6	65.33	66.80		
	L	5 Hz	9	28.00	17.14	0.90	ns	10	35.60	43.45	0.38	ns	8	28.25	13.66	1.04	ns	7	71.85	64.14	0.42	ns

(*z* value) Mann-Whitney *U* analyses; ns: not statistically significant; R: right; L: left, mAmp: milliamperes.

AO: atypical odontalgia, TN: trigeminal neuralgia, M: migraine, TMD: temporomandibular disorder.

**Table 6:** Comparison of the current perception threshold (CPT) between groups.

	AO			TN			M			TMD		
	2000 Hz	250 Hz	5 Hz	2000 Hz	250 Hz	5 Hz	2000 Hz	250 Hz	5 Hz	2000 Hz	250 Hz	5 Hz
Pain-free	3.07**	3.01**	2.64**	1.87	1.93	2.8**	0.14	1.22	1.45	0.72	0.13	0.36
AO				0.71	0.47	1.35	3.61***	1.67	1.03	1.62	1.49	0.95
TN							1.69	0.84	1.54	1.46	0.83	1.78
M										0.39	0.76	0.97

(*z* value of Mann-Whitney  *U* analyses) *P < 0.05; **P < 0.01; ***P < 0.001.

AO: atypical odontalgia; TN: trigeminal neuralgia; M: migraine; TMD: temporomandibular disorder.

**Table 7:** Differences between groups in psychopathological and aggressive behavior dimensions.

		AO/PF	TN/PF	M/PF	TMD/PF	AO/TN	AO/M	AO/TMD	TN/M	TN/TMD	M/TMD
	Assault										
	Indirect hostility	−2.08*		−2.17*							
	Irritability			−2.69**							
	Negativism										
BDHI	Resentment	2.75**				1.91*	3.58***	2.86**			
	Suspicion		2.18*			−2.82**			3.36***	3.17**	
	Verbal hostility										
	Guilt								1.99*		
	BDHI total									2.02*	

	Depression	2.63**	2.75**		2.31*			2.62**			
IDAS	Anxiety						3.0**				
	Irritability								2.08*	2.08*	

	Somatization		3.00**	2.48*	3.59***	−2.20*		−2.38*			
	Obsessive compulsive		2.45*								
	Interpersonal sensitivity					−2.11*			1.96*		
	Depression										
SCL-90-R	Anxiety							−2.06*			
	Hostility										
	Phobic anxiety										
	Paranoid ideation		2.11*								
	Psychoticism	2.45*									
	SCL-90-R total scoring		2.04*								

(*z* value of Mann-Whitney analyses) *P < 0.05; **P < 0.01; ***P < 0.001; ****P < 0.0001.

AO: atypical odontalgia; TN: trigeminal neuralgia; M: migraine; TMD: temporomandibular disorder; PF: pain free; BDHI: Buss-Durkee hostility inventory; IDAS: irritability depression anxiety scale; SCL-90-R: Symptom Checklist 90 Revised.

**Table 8:** Correlation between current perception threshold measures and behavioural and psychopathological dimensions.

		AO (*n* = 18)			Total sample (*n* = 76)			Other pain syndromes (*n* = 46)		
		2000 Hz	250 Hz	5 Hz	2000 Hz	250 Hz	5 Hz	2000 Hz	250 Hz	5 Hz
BDHI	Resentment	−0.62*	−0.53*	−0.54*	0	−0.04	−0.04	0.30*	0.17	0
IDAS	Depression	−0.39	−0.15	−0.52*	−0.05	−0.01	−0.16	−0.04	−0.03	−0.26

(Spearman rho coefficient) *P < 0.05; **P < 0.01; ***P < 0.001; ****P < 0.0001.

BDHI: Buss-Durkee hostility inventory; IDAS: irritability depression anxiety scale; AO: atypical odontalgia.

**Table 9:** The association of current perception threshold (CPT) and psychopathological dimensions with axis I and axis II psychiatric disorder.

		*n*	CPT	CPT	BDHI	BDHI	SCL-90-R	SCL-90-R	SCL-90-R	IDAS	IDAS
			A*β*	C	resentment	suspicious	depression	somatization	hostility	depression	anxiety
DSM		74									
Axis I	Current major depressive episode	10	−2.64**	−2.08*	2.01*					3.28***	1.97*
	Avoidant	13	−2.59**		2.20*	2.69**	3.79****		4.31****	2.69**	3.31***
Axis II	Obsessive compulsive	20	2.81**								
	Paranoid	14				3.23***	2.66**	2.29*	2.99**		

*z* values of Mann Whitney analysis; *P < 0.05; **P < 0.01; ***P < 0.001; ****P < 0.0001.

AO: atypical odontalgia; TN: trigeminal neuralgia; M: migraine; TMD: temporomandibular disorder; PF: pain free; BDHI: Buss-Durkee hostility inventory; IDAS: irritability depression anxiety scale; SCL-90-R: Symptom checklist 90 revised; DSM: diagnostic statistical manual.
